# Differential costs for the non-adult ADHD population in Catalonia

**DOI:** 10.1186/s13561-023-00437-8

**Published:** 2023-04-22

**Authors:** Toni Mora, Jaume Puig-Junoy, Rowena Jacobs, Jordi Cid

**Affiliations:** 1 Research Institute for Evaluation and Public Policies (IRAPP), Universitat Internacional de Catalunya (UIC), Barcelona, 08017 Spain; 2 Universitat Pompeu Fabra-Barcelona School of Management (UPF-BSM), Barcelona, Spain; 3 Centre for Health Economics (CHE), University of York, York, UK; 4 Institut d’Assistència Sanitària (IAS) and Mental Health & Addiction Research Group (IDIBGI), Girona, Spain

**Keywords:** ADHD, Non-adult population, Economic burden, Cost-of-illness, I10, I14

## Abstract

Attention-Deficit/Hyperactivity Disorder (ADHD) is young children’s most common mental health disorder. We aim to provide causal estimates of the differential costs for the non-adult population with ADHD. We used longitudinal administrative data covering the non-adult population over five years and different healthcare providers (general practitioners, hospitalisations and emergency departments, visits to mental healthcare centres—day-care or hospitals) of 1,101,215 individuals in Catalonia (Spain). We also include the consumption of pharmaceuticals and cognitive therapies. We instrumented ADHD diagnosis by the probability of being diagnosed by the most visited healthcare provider based on individual monthly visits to the provider in which this visit was related to ADHD and the density of professionals in the different mental health providers. After using matching procedures to include a proper control group, we estimated two-part and finite mixture models. Our results indicate that ADHD children and adolescents displayed 610€ higher annual health direct costs compared to not diagnosed counterparts. We provide average costs disentangling the sample by age boundaries, gender, and comorbidities to offer values for cost-effective analyses and incremental costs after diagnosis, which is around 400€. A significant differential annual direct health cost for the non-adult population with ADHD is determined, which will be helpful for cost-effectiveness analysis and complete cost-of-illness studies.

## Introduction

Attention-Deficit/Hyperactivity Disorder (ADHD) is young children's most common mental health disorder. Prevalence rates worldwide range from 3 to 7% (average of around 5%) of school-aged children [1], and these rates are increasing over time. ADHD is a childhood-onset neurodevelopmental disorder with hyperactivity, impulsivity, and inattention as core symptoms [2]. A global number of prevalent ADHD cases has been recently estimated [3] in thousands in 2019 of 84,709.0 (95% CI: 62,461.8– 111,261.9), and a global age-standardised prevalence per 100,000 (all population) of 1,131.9 (95% CI: 831.7– 1,494.5).

This disorder has a functional impact on children and adults [4]. The natural history of ADHD shows that an essential proportion of patients, around 65%, continue to meet full criteria or partial remission of symptoms by adulthood [5].

For these reasons, studies of healthcare costs are essential to help policymakers to plan mental health services and treatment. It is well known that the non-adult population with ADHD consumes more healthcare resources than those without ADHD [6]. However, little is known about this circumstance in Europe. In this regard, we contribute to calculating the public direct differential health cost of an ADHD population cohort from Catalonia. We use extensive administrative data covering the non-adult population over five years and different healthcare providers (GPs, hospitalisations and emergency departments, visits to mental healthcare centres, day-care or hospitals). We also consider the consumption of pharmaceuticals and psychological therapies controlled through an extensive list of comorbidities.

The main contribution of this paper is to provide causal estimates of the differential costs for the non-adult population with ADHD instrumenting ADHD diagnosis by the probability of being diagnosed by the most visited healthcare provider based on individual monthly visits to the provider in which this visit was related to ADHD and the density of professionals in the different mental health providers. That is, we use supply information to identify these differential healthcare costs correcting our estimates by instrumental variables (IV) to avoid the influence of confounders. For this purpose, we use the whole population in Catalonia, both those diagnosed with ADHD and those without, and provide average annual costs for different subgroups of the population disaggregated by age and gender, in addition to considering some comorbidities that might induce higher costs. Further, we provide information about incremental costs after being diagnosed and heterogeneity according to adherence to medication.

## Related literature

Cost analyses differ greatly depending on the country analysed, treatment choice, and the database used [7–9], although most research originates from the United States. Indeed, annual medical costs differ depending on whether paired controls are higher for those diagnosed with ADHD than undiagnosed (503$ to 1,343$) or unchecked (207$ to 1,560$).

The limited number of European studies examining the economic burden of ADHD highlights the need for research in this area. A systematic review [10] pointed out estimated per-person differences for the Netherlands. The authors pointed out that direct healthcare costs ranged between 798€ and 3,571€. This study provides a comprehensive estimate of the social and healthcare costs of ADHD. Despite uncertainties due to the small number of studies identified and the wide range of cost estimates, the results suggest that ADHD imposes a significant economic burden on multiple European public sectors. Another research project [11] used a German database and randomly selected an age-appropriate ADHD-free reference group matched to the 25,300 individuals diagnosed with ADHD. Total health and ADHD-related care costs were analysed, and more specific analyses of ADHD comorbidities in adulthood (substance use disorders, anxiety, and mood and obesity disorders). The costs of comorbidities in people with ADHD were calculated concerning the costs of these same conditions in people without ADHD.

A recent systematic review of the global economic burden of ADHD [12] estimated total costs ranging from $US831 to 20,538 per person; also, estimates based on marginal costs (excess or attributable costs of ADHD) ranged from $US244 to 18,751 per person.

Interestingly, costs increase with age. Subgroup analyses were performed by age (0–12 years, 13–17 years, 18–30 years, over 30 years) and sex. This project [11] concluded that the excess costs of care for patients with ADHD (compared to the costs of care for people without ADHD) amount to 1,549€ per year for women and 1,467€ for men (the average is calculated for all age groups).

Other studies compare costs before/after diagnosis. In this regard, patients with ADHD show higher resource utilisation compared to a control group [13], even before diagnosis. This was recognised by the higher costs of the year before diagnosis compared to the control group and the increased use of methylphenidate and atomoxetine (pharmacological treatment). These authors estimate higher costs of about 1,006€ a year after diagnosis. In this regard, children with ADHD have more medical expenses than similar children without ADHD [14]. This excess cost precedes the initial diagnosis of ADHD by at least two years, indicating the presence of problems before the initial diagnosis. The extra costs are even higher once diagnosed, mainly due to increased psychiatric and paediatric care and medication costs. These authors get a differential of 1,328$ in the first year and 1,040$ in the second year. Before diagnosis, it should be noted that the costs were already 678$ higher for children and adolescents diagnosed with ADHD. Strictly related to the Spanish context [15], a project descriptively used a sample of 321 children and adolescents with ADHD from 15 health units and evidenced that total direct costs were 5,733$ per year in 2012 prices, mostly related to psychological or educational support. Indeed, direct costs accounted for 60.2% of the total costs (€3,450), whilst support from a psychologist/educational psychologist represented 45.2% of direct costs and 27.2% of total costs, respectively. Likewise, pharmacotherapy accounted for 25.8% of direct costs and 15.5% of total costs. Finally, among indirect costs (€2,283), 65.2% was due to caregiver expenses.

However, there are many limitations concerning the abovementioned studies for different reasons: calculations from averages, preliminary designs, biased samples, use of cohorts with ADHD, databases related to US insurers, lack of detailed clinical information, the inclusion of comorbidities or even more critical, non-consideration of appropriate controls. These limitations, such as not including other comorbidities, will bias the differential effect of ADHD on individual costs. We seek to address some of these limitations in this study.

## Methods

### Data sources and study population

The Ethical Review Board approved the study in Hospital Trueta & IAS, Girona (Spain). We use a large administrative dataset from The Agency for Health Quality and Assessment of Catalonia that includes information from several providers, although considering different periods for the whole population of Catalan children born between 1998 and 2012, including those diagnosed with ADHD and those not being diagnosed. We focussed on cohorts of children over six years old because they are unlikely to get an ADHD diagnosis before this age (1,101,215 individuals).

This database contains information on primary care, hospitalisations, emergency care, mental health hospitalisation, and community mental health care from 2013–2017. It also encompasses an individual identifier, the visit date (length in case of hospitalisations), and all diagnoses and procedures registered in these visits. The International Classification of Diseases, ninth revision, Clinical Modification (ICD-9-CM) diagnostic manual is used for diagnostic purposes in Catalonia. Diagnoses are shown in an ordinal sense, indicating the primary diagnosis for that visit and a list of secondary diagnoses. Via unique personal identifiers, the information is linked between all provider's datasets but also to some demographic information: gender, age, drug co-payment level, which is related to the socioeconomic status of their parents, individual nationality, date of death and the sanitary health region they belong to.

There are 2,800 healthcare procedures (HCP) in the dataset defined and classified according to the ICD-9-CM. The unit cost of each HCP has been imputed using a complete list of public prices approved in 2013 by the Department of Health in Catalonia (Spain) for the Catalan Healthcare Service for primary care services, hospital and specialised services, and psychiatric and mental health.

### Costs dataset

No list of public prices has been officially published for Catalonia since 2013. However, after 2013, unit prices for hospital and specialised visits were actualised or incorporated until 2020. However, very few prices for healthcare procedures have been included. Therefore, the approved public prices for 2013 are used as the leading resource to impute the cost of the HCP. Public prices approved in 2013 for primary care include unit prices or tariffs for standard primary care services such as GP visits, ambulatory care, or domiciliary care. In the case of hospitals and specialised services, most of the prices for HCP are set according to the Diagnostic Related Group (DRG), including prices for a wide range of surgical procedures, implants, infections, plastic surgery, etc. Other hospital and specialised services tariffs include rehabilitation and physiotherapy prices, laboratory test prices, and further tests, procedures, and therapies undertaken as part of the primary diagnosis.

Prices set by the Department of Health in Catalonia for each DRG vary according to the group of hospitals they are paid to. The Department distinguishes four groups of hospitals and classifies each Catalan hospital into one of these four groups according to the resource used and their structural capacity (i.e., number of beds). Therefore, for the same DRG, the Department of Health sets a different price according to each group of hospitals: a unified price for group 1 (isolated basic general hospitals and complementary hospitals) and 2 (basic general hospitals), a price for those hospitals belonging to group 3 (referral hospitals), and a price for those classified in group 4 (high technology hospitals and high technology and specialist hospitals). There are 72 hospitals in the dataset, and information is provided on which hospital individuals have been treated every time they require hospital care. Therefore, it is possible to identify which group each belongs to and impute the corresponding DRG costs established for that group of hospitals. We show in Table A1 in the Appendix the considered tariffs for the analysed period.

However, the Catalan Health Service does not provide a tariff for all healthcare procedures. In such a case, the DRG price is assigned where a healthcare procedure may belong and be performed. As explained above, since groups of hospitals set prices for DRGs, HCPs will have different prices according to the hospital where they performed. Moreover, if an HCP occurs in more than one DRG, the average across all the possible DRGs by groups of hospitals is taken. Therefore, the price inputted for a particular HCP can be either a special tariff, differing by a group of hospitals, or an average of different DRG prices. If neither the unit price nor the DRG price by the hospital’s group is found for Catalonia, the price of healthcare procedures is approximated by searching for prices of other regions in Spain in any available year. The platform Esalud has used an online and up-to-date database of reported Spanish healthcare costs. Prices in Esalud are deflated using either the 2018 or the 2019 consumer price index. Therefore, to homogenise prices, we input the actualised price. If the price for an HCP is found for more than one Spanish region, we average their corresponding actualised prices. Overall, 93% of the prices have been found, where 66% come from a single tariff (either from Catalonia or another Spanish region) and 27% come from DRGs and groups of hospitals. Since the dataset encompasses HCPs from 2013 to 2017, imputed prices are deflated to 2017, using the corresponding Consumer Price Index for each year (see: https://www.ine.es/prensa/ipc_tabla.htm. Instituto Nacional de Estadística).

Drug costs were considered from the funder's perspective, not the total consumer price after co-payment. Individuals with costs above the 99th percentile have been removed, as they may be cases of severe illness (outliers). We further excluded those individuals whose total average yearly costs were above the 95^th^ percentile. These individuals may distort average behaviour and are related to serious illness cases.

Finally, we compute the differential and incremental costs. The differential cost is defined as the additional annual cost that an individual with ADHD faces in the national health service. In contrast, the incremental cost is the annual cost that an individual with ADHD experiences during the period after diagnosis compared to the period before the first diagnosis.

### Econometric methodology

To compute the differential cost of a non-adult individual with ADHD, we must consider two relevant factors: (i) the econometric technique to be used and the control units, and; (ii) the factors associated with higher costs to be included in the regressions and the inclusion of specific comorbidities. The first factor is the asymmetric distribution of the variable costs and the methodology's specific bimodality condition. We have estimated it using different alternatives: neighbourhood matching (considering at least five individuals with the same characteristics: gender, age boundary, asthma comorbidity and the quarter of birth), propensity score matching, and two-part models. All of these models provide complementary approaches. The first two methods focus on finding units that act as a control (the overall population does not constitute a control group per se). At the same time, the third one considers the estimation of the incremental cost in the two parts of the distribution (low costs as opposed to high costs). The latest model has also been estimated considering the individuals identified as controls using the neighbourhood matching technique for robustness. This technique allows us to run through the list of treated units and select the closest eligible control unit to be paired with each treated unit for a homogeneous comparison. Finally, we also considered estimating using finite mixture models (FMM), which usually accommodate the case of costs better given their high asymmetry and the presence of individuals with high costs that complicate average comparisons. FMM is useful to model the probability of belonging to each unobserved group (non-cost, low use and heavy use) to estimate distinct regression model parameters in each group. We considered two latent classes to explain the different distribution parts in this case. For our approach, the first part of the positive costs is relevant, that is, the one not affected by heavy users or healthcare resources.

Our identification strategy is based on instrumenting ADHD diagnosis by the probability of being diagnosed by the most visited healthcare provider based on individual monthly visits to the provider in which this visit was related to ADHD (*prop*_*i*_) [16]. The instrument is associated with the probability of being diagnosed based on the healthcare provider characteristics that might condition an excessive (or low) proportion of becoming diagnosed after conditioning by individual and parental features. At the same time, as a second instrument, we use a measure associated with the density of professionals in the different mental healthcare providers [17]. This total number of mental health professionals is divided by the reference population (*prof*_*i*_). Once the most frequented supply unit has been identified, this ratio of professionals is assigned. The rationale behind this second instrument is that a higher supply of professionals could reduce those not correctly diagnosed and thus reduce the percentage of those diagnosed [18]. This step is shown in Eq. (2). Again, we consider all health provider units present in Catalonia instead of using only the units of mental healthcare providers. Equation (1) shows the equation to be estimated to explain the total medical costs.

1 $${Y}_{i}={X}_{i}\beta +{diag}_{i}{\gamma }_{1}+{abs}_{i}+{\varepsilon }_{i}$$

2 $${diag}_{i}={prop}_{i}+{{prof}_{i}+X}_{i}\delta +{u}_{i}$$

where *Y*_*i*_ indicates the average of the total direct health costs of the individual pooled during the period 2013–2017 and, while *X*_*i*_ is a set of observable characteristics (sex, age, nationality, trimester of birth, co-payment rates and out-of-pocket limits per person) and some comorbidities at the level of the year such as the existence of visits due to: overweight, asthma, learning disability, depression, anxiety, conduct disorder and challenging disorder. Age was non-linearly introduced. Copayment levels constitute an appropriate proxy for income levels, given that copayment percentages are defined based on parental income levels [19]. *diag*_*i*_ is a dummy variable taking the value one if an individual is diagnosed. We include fixed effects that may affect prescribing, i.e., basic health areas for the health sectors (*abs*_*i*_). Models also considered error terms (*ε*_*i,t*_). Both equations were estimated simultaneously, and standard errors were clustered at the *abs* level because most healthcare decisions and experiences are shared at this aggregate level. Akaike information criteria showed that the two-part model was the best estimation procedure.

## Results

### Descriptive statistics

**Fig. 1:**
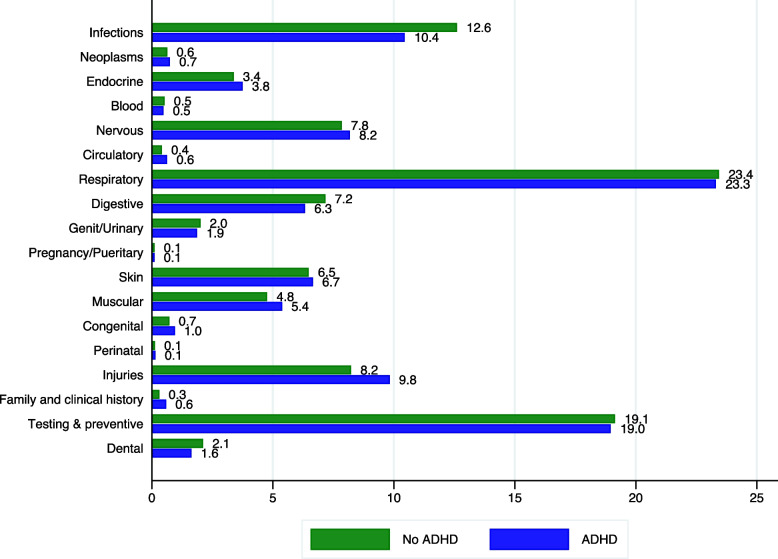
Aggregated diagnoses groups over the period 2013–2017 based on ADHD diagnosis. Note: We excluded the groups related to mental diseases

Table 1 shows the demographic characteristics of diagnosed ADHD individuals compared to the overall population. ADHD children are older, and the percentage of male and Catalan individuals with Spanish nationality is consistently higher. Likewise, based on parental income levels, co-payment levels illustrate that diagnosed are wealthier than the average population (as proxied by higher co-payments). Figure 1 shows differential prevalence rates when comparing aggregate diagnoses groups for individuals with ADHD relative to those not diagnosed with ADHD. ADHD children show statistically significant dissimilar percentages in some comorbidity groups over 2013–2017. Consequently, comparing ADHD-diagnosed children with comparable individuals is crucial when estimating cost differences.

**Table 1:** Socioeconomic characteristics differences in ADHD diagnosis

	**ADHD population** **(*N*=45,385)**	**Overall population without ADHD** **(*N*=1,080,881)**	**Matched group** **(*N*=84,576)**
*Average age*	12.17 (3.35)	11.01 (3.70)	11.54 (3.30)
*Female*	0.28 (0.44)	0.49 (0.50)	0.44 (0.50)
*Spanish*	0.94 (0.22)	0.79 (0.41)	0.88 (0.33)
*Date of birth*			
*1*^*st*^* quarter*	0.20 (0.40)	0.24 (0.43)	0.23 (0.41)
*2*^*nd*^* quarter*	0.23 (0.42)	0.25 (0.43)	0.23 (0.44)
*3*^*rd*^* quarter*	0.27 (0.44)	0.26 (0.44)	0.26 (0.42)
*4*^*th*^* quarter*	0.30 (0.46)	0.25 (0.44)	0.28 (0.43)
*Exempted*	0.05 (0.22)	0.04 (0.21)	0.04 (0.20)
*10% co-payment*	0.09 (0.29)	0.05 (0.22)	0.05 (0.21)
*40% co-payment*	0.54 (0.50)	0.58 (0.49)	0.55 (0.48)
*50% co-payment*	0.30 (0.46)	0.29 (0.45)	0.32 (0.46)
*60% co-payment*	0.02 (0.12)	0.01 (0.12)	0.02 (0.11)
*Excluded from co-payment*	0.00 (0.07)	0.03 (0.17)	0.02 (0.16)
*Asthma (83,080)*	10.38%	6.42%	6.84%
*Learning disability (34,111)*	16.27%	1.91%	3.69%
*Depression (4,161)*	1.00%	0.24%	0.41%
*Anxiety (50,485)*	10.36%	3.50%	5.21%
*Conduct disorder (39,087)*	13.85%	2.34%	4.75%

Standard deviation in parenthesis

**Fig. 2:**
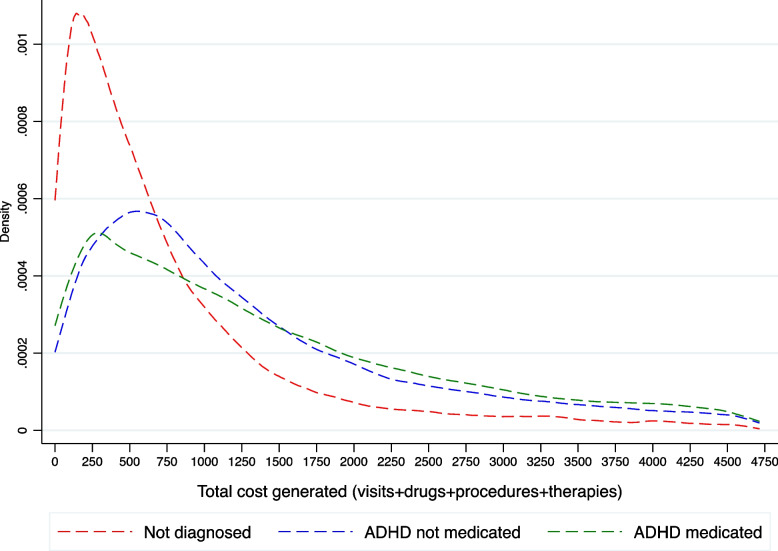
Annual costs distributions based on diagnosis and medication status. Note: we plot costs below percentile 90. Total costs include visits (to GPs, hospitalisations and emergency departments, visits to mental healthcare centres -day-care or hospitals), drugs consumption, healthcare procedures and cognitive therapies

**Fig. 3:**
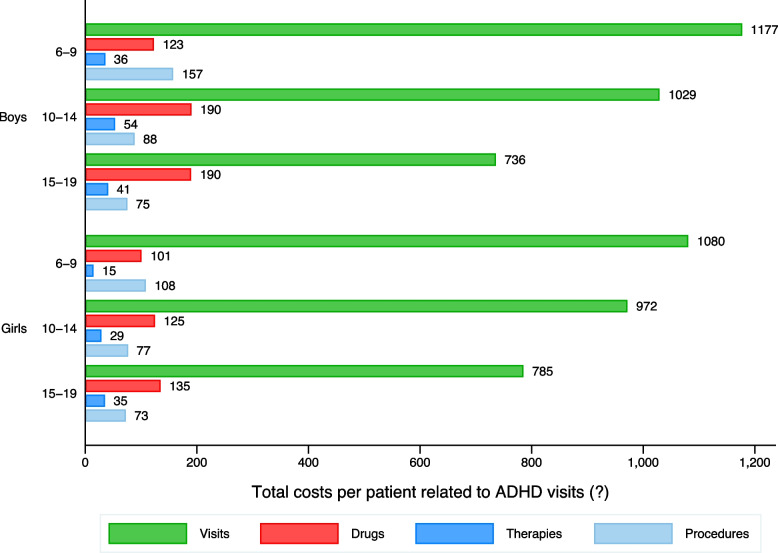
Relevance of kind of costs per patient within ADHD across gender and age (overall period)

We observe that the cost distribution is not strictly comparable across all individuals. Figure 2 provides evidence that the distribution of costs is different in those diagnosed but not medicated compared to those diagnosed and medicated. Since we have access to the whole population, we compute total costs and healthcare resources for individuals with some related mental comorbidities and asthma. For the latter, international comparisons of the most prevalent conditions tend to use asthma as a reference, given its prevalence rate [20]. Here, we provide total costs generated by individuals with some clinical diagnoses, not the exclusive costs derived from these specific comorbidities. Table 2 illustrates that overall costs are higher for the ADHD population after considering sociodemographic characteristics and some comorbidities such as learning disability, anxiety and conduct disorder. We also provide average costs (1,294.4€), disaggregated using age boundaries, gender, and comorbidities to offer values for cost-effectiveness analyses. Significant differences are present after computing average costs once some comorbidities are present such as depression (1,931.74€) or defiant disorder (1,870.77€). We also have calculated median values for the direct healthcare costs, given the skewed distributions that costs usually show. However, these differences are not too dissimilar to the average values. Figure 3 shows the percentage of all per-patient costs amongst those diagnosed with ADHD and reveals significant differences across age boundaries and gender. Most costs are related to visits but show an increasing pattern of drug expenditure prescription trends as individuals become older.

**Table 2:** Average annual costs (€) with ADHD condition

	**Average cost (std. dev.)**	**Median cost (CI 95%)**
*Population*	1,294.38 (994.11)	984.66 (974.3 – 994.57)
*Male*	1,314.76 (1,010.24)	1,010.24 (998.03—1,021.88)
*Female*	1,240.90 (918.93)	918.93 (900.11—939.20)
*Age boundary*		
*6–9 yo*	1,239.18 (902.43)	902.43 (870.33—935.22)
*10–14 yo*	1,350.11 (1,033.74)	1,033.74 (1,016.25—1,050.78)
*15–19 yo*	1,260.22 (965.67)	965.67 (952.60—981.82)
*Overweight*	1,486.99 (1,177.99)	1,177.99 (1,137.19—1,213.72)
*Learning disability*	1,537.87 (1,260.27)	1,260.27 (1,236.30—1,294.85)
*Depression*	1,931.74 (1,786.64)	1,786.64 (1,670.32—1,933.95)
*Asthma*	1,588.83 (1,350.23)	1,350.23 (1,314.13—1,387.47)
*Anxiety*	1,642.86 (1,360.37)	1,360.37 (1,312.80—1,411.43)
*Conduct disorder*	1,681.32 (1,439.13)	1,439.13 (1,406.64—1,479.10)
*Defiant disorder*	1,870.77 (1,667.17)	1,667.17 (1,599.14—1,743.94)

Standard deviation in parenthesis. Individuals older than six years old. We excluded outliers (costs above the 95^th^ percentile)

### Econometric results

Table 3 shows the results of estimating cost differences using alternative estimation procedures and our instrument selection. The best fitting performance was obtained for the two-part model using matched individuals as a control group and the FMM estimation, which only accounts for positive costs. Although the two-part model outperformed the other econometric approaches, results were similar, and IV estimations did not change baseline estimates. Our results indicate that ADHD children and adolescents had 609.8€ (593.2€-626.3€) higher annual health direct costs compared to not diagnosed counterparts (ADHD incremental average cost). Table A2 in the Appendix shows the complete list of estimated coefficients for the rest of the covariates. This finding was confirmed after accounting for only positive costs through the FMM procedure. Considering two classes, the differential health direct costs for normal users was 757.7€ (753.4€-761.9€), whereas the difference for heavy users of healthcare resources was 420€ (418.5€-421.6€). We also ran a separate model for males and females. Males showed a cost difference of 655.5€ (638.3€-672.6€), whereas females’ results indicated a 581.5€ difference (559.8€-603.3€).

**Table 3:** Differential annual costs: alternative estimation results

	**IV estimation**
*Matching neighbouring*	693.71 (26.25)^c^
*Propensity score matching*	634.26 (10.71)^c^
*Two-part model*	632.27 (8.24)^c^
*Two-part model (matched units)*	609.75 (8.45)^c^
*FMM 2 latent class (matched units) for positive costs*	
*Class1*	757.67 (2.18)^c^
*Class2*	420.04 (0.80)^c^

^a^, ^b^ and ^c^ denote statistical significance levels 10%, 5% and 1%, respectively. The sample size is 967,684; 967,638; 767,099; 84,995; and 124,720, respectively. Standard errors are in parenthesis. Standard errors for FMM estimations are based on mean differences across predictions for both classes. Class 1 refers to regular healthcare users, whereas Class 2 identifies heavy healthcare users after dropping those individuals with high costs. All regressions include as controls: the quarter of birth, age, gender, nationality, and the considered comorbidities (overweight, learning disability, depression, asthma, anxiety, conduct disorder, and defiant disorder), copayment levels plus pharmacy limits and basic health areas fixed effects

Measuring potential over-diagnosis or improper classification of ADHD is essential. We established several definitions of children diagnosed with ADHD: (i) those diagnosed with the ICD-9 codes related to ADHD in any healthcare provider, (ii) children consuming drugs related to the disease, and (iii) a classification informed by clinical expertise. Specifically, concerning the latter, we categorised children into three groups: (i) highly likely ADHD diagnosis, (ii) potentially likely ADHD diagnosis, and (iii) not very likely ADHD diagnosis. A highly likely ADHD diagnosis occurs when we find a principal ADHD diagnosis in mental health centres or inpatient mental health units from the comprehensive Catalan public health system (SISCAT). Potentially likely ADHD diagnosis is when we identify an ADHD pharmacological treatment and secondary ADHD diagnosis in mental health centres or inpatient mental health units or ADHD diagnosis in primary care (we identified those patients who were diagnosed and treated by private practice, although they might have benefitted from public pharmacological treatment). Then, we restricted our analysis to those individuals classified as highly likely ADHD diagnosis or potentially likely ADHD diagnosis to avoid overdiagnosis-related concerns. In this case, our results revealed a higher difference of 779.6€ (749.9€-809.2€) than the one obtained using the non-specific definition (609.8€). For those potentially likely, the results evidenced a higher difference of 585.3€ (564.4€-606.3€).

### Incremental cost after being diagnosed

Next, we evaluated the cost differences between the year before and the years following the first diagnosis. We report these figures through this section. In this case, we compare the total expenditure, again eliminating extreme cases, in the annual period before the first diagnosis and comparatively annually up to five years after that diagnosis only for children and adolescents with ADHD. Descriptively, the increase is evident in the first year after diagnosis (392.2€), and from that year, some downward stabilisation to 380.1–385.3€ seems evident. However, we adjust these differentials compared to the annual cost before diagnosis, conditional on the characteristics of the individuals. Again, the distributions are asymmetric, so estimates using ordinary least squares and GLM (generalised linear models) have been compared. Comparing the fitting performance of these models using the Akaike criterion, the GLM estimate shows the best results. Thus, compared to the initial period and adjusting for the characteristics of the individuals, the costs are higher: 413.3€ (385.5€-441.1€) after one year, 401.1€ (376.2€-426.0€) after two years, 371.6€ (347.8€-395.3€) after three years, 365.4€ (341.9€-388.9€) after four years and 366.9€ (342.0€-391.8€) after five years.

### Cost differential accounting for adherence

We also account for the impact of adherence on the differential cost but exclude ADHD drug costs from overall costs (direct non-pharmaceutical costs). We measured adherence to ADHD drugs by computing either the medication possession ratio (MPR) or the proportion of days covered (PDC). These are standard measures to compute adherence. Additionally, we used a dummy representing those with complete adherence to ADHD medication. Results are shown in Table 4. The differential cost was reduced to 404.9€ (353.6€-456.2€), but those showing adherence significantly differed in total costs by 829.6€ although this was highly heterogeneous (178.1€-1,481.2€). The latter is related to the fact that these individuals consumed other drugs more frequently or showed more visits, which is corroborated in Figure A1 in the Appendix.

**Table 4:** Differential annual costs accounting for adherence and total costs except costs related to ADHD drugs

	**Two-part IV estimation**
*ADHD condition*	285.84 (58.74)^c^
*Adherence (MPR)*	785.67 (323.44)^b^
*ADHD condition*	296.58 (54.08)^c^
*Adherence (PDC)*	804.69 (323.88)^b^
*ADHD condition*	404.86 (26.18)^c^
*Dummy full adherence (either MPR or PDC)*	829.64 (332.42)^b^

^a^, ^b^ and ^c^ denote statistical significance levels 10%, 5% and 1%, respectively. Standard errors are in parenthesis. The sample size is 81,770 which refers to those fully adherent to medication. All regressions include as controls: the quarter of birth, age, gender, nationality, and the considered comorbidities (overweight, learning disability, depression, asthma, anxiety, conduct disorder, and defiant disorder), copayment levels plus pharmacy limits and basic health areas fixed effects

## Discussion

This study estimated the differential direct healthcare costs of the non-adult population with ADHD in Catalonia (Spain). Indeed, we contribute to the scarce literature within the European context on ADHD costs. More importantly, no previous studies have assessed the economic impact of ADHD on the utilisation of healthcare resources accounting for longitudinal administrative data and using a method that allows us to identify causal differential healthcare direct costs conditional on an extensive list of comorbidities that might alter this differential effect (ADHD incremental cost). Likewise, to measure resource consumption, we considered a complete list of public healthcare providers (GPs, hospitalisations and emergency departments, visits to mental healthcare centres—day-care or hospitals), including all relevant resources (visits, procedures, pharmacological consumption, and other therapies).

According to previous research [11], our data corroborated that ADHD with comorbidities (including mental illness) has a higher average cost than ADHD alone. We also found a heterogeneous incremental annual cost within the groups of non-ADHD, not medicated ADHD, and ADHD-medicated according to age and sex, as shown in previous research [10, 14]. ADHD average annual costs per person in Catalonia (1,294.4€) are in the lower band of direct costs relative to international levels [12], although health systems are not fully comparable. Estimations using the two-part model regression after matching individuals with ADHD with those without ADHD outperformed other models. Our findings point to 609.8€ higher annual health direct costs compared to non-diagnosed counterparts. We also provide average costs disaggregated using age boundaries, gender, and comorbidities to offer values for cost-effectiveness analyses. This is the first time that we have data on the differential costs of ADHD treatment in children and adolescents when compared with an equivalent group without the disorder in Catalonia.

One of our study's limitations is that we could not consider private use healthcare costs and costs related to diagnostic tests and referrals. This information is still not available. Additionally, we can’t disentangle which type of ADHD was predominant within ADHD-diagnosed individuals because the diagnosis codes at that date did not differentiate this issue. Next, ICD-9 was the criteria implemented in Catalonia during the analysed period. We acknowledge that a different standard, such as the Diagnostic and Statistical Manual of Mental Disorders (DSM), could have resulted in a different prevalence of the non-adult ADHD population and affected our differential cost findings. For this reason, we have included a correction that might avoid potential overdiagnosis impacting direct health costs related to the non-adult ADHD population. Finally, the consumer price index was a limitation, given that an appropriate public health services price index is not allowable in Spain.

The data on the use of services in treating ADHD showed heterogeneity in services and treatment pathways. If improvements are introduced to reduce the variability of clinical practice in treating ADHD, having this cost reference value will allow us to monitor costs and compare them with a previous reference value. This can contribute to the continuous improvement of ADHD treatment care planning. Finally, incremental costs after diagnosis are estimated at around 400€. This cost estimate highlights the importance of early detection of ADHD to reduce future healthcare costs.

## Figures and Tables

**Fig. 4:**
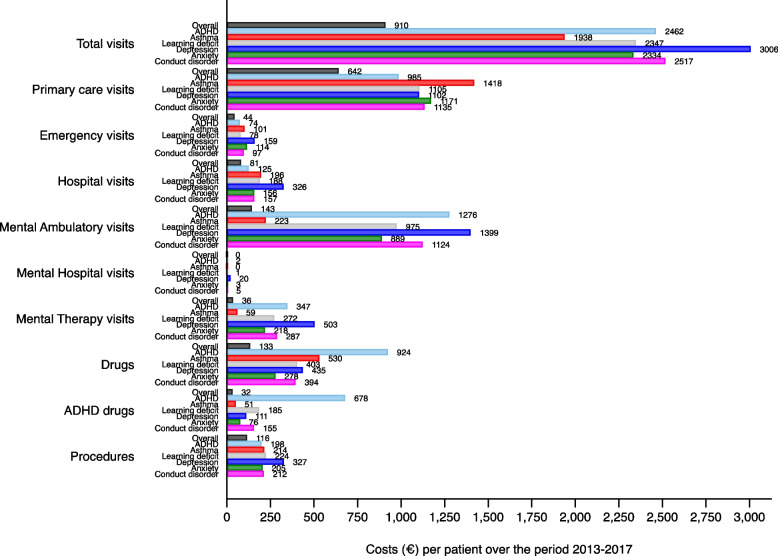
HCU costs per patient over the period 2013–2017 for several diseases

## Data Availability

The Agency for Health Quality and Assessment of Catalonia (AQuAS) supplied data under an agreement that does not allow researchers to share the database with any other third party.

## Appendix

**Table 5:** List of tariffs based on kind of visit per year

Visits	2013	2014	2015	2016	2017
*Primary Care*					
Not urgent visit at a primary care centre	41	41	41	41	41
*Hospitalisation*					
Complementary hospital	773.7	773.7	773.7	787.4	792.3
General basic A hospital	895.8	895.8	895.8	911.7	917.4
General basic B hospital	967.1	967.1	967.1	984.2	990.4
Reference A hospital	1,191.1	1,191.1	1,191.1	1,212.1	1,219.7
Reference B hospital	1,623.7	1,623.7	1,623.7	1,652.4	1,662.8
High complexity hospital	2,046.2	2,046.2	2,046.2	2,082.4	2,095.5
Specialised hospital	2,046.2	2,046.2	2,046.2	2,082.4	2,095.5
*Emergency room*					
Triage	15.3	15.3	15.3	15.5	15.6
Attended urgency	86.5	86.5	86.5	88.1	88.6
*Mental health hospital (hospitalisation tariffs)*					
Hospitalisation: acute	174.9	174.9	178.0	181.2	182.3
Hospitalisation: subacute	110.0	110.0	112.0	114.0	114.7
Dual pathology unit	174.0	174.0	177.2	180.3	181.4
Ambulatory mental (referrals)	157.8	159.6	161.4	163.3	165.2

Own source from public official administrative diaries

**Table 6:** Complete list of estimated coefficients: two-part model matched units (IV estimation)

	2PM IV marginal effects
*ADHD condition*	609.75 (8.46)^c^
*born 2nd quarter*	-16.04 (6.33)^b^
*born 3rd quarter*	-9.13 (6.65)
*born 4th quarter*	-10.84 (6.52)^a^
*Age*	-6.25 (0.91)^c^
*Female*	-40.95 (4.84)^c^
*Spanish*	77.32 (8.68)^c^
*Overweight*	145.95 (10.28)^c^
*Learning disability*	299.38 (12.19)^c^
*Depression*	603.14 (37.15)^c^
*Asthma*	350.67 (9.39)^c^
*Anxiety*	407.27 (11.75)^c^
*Conduct disorder*	401.00 (12.95)^c^
*Defiant disorder*	483.23 (31.57)^c^
*Pharmacy limit Up to 8.23€*	32.21 (15.40)^b^
*Pharmacy limit Up to 18.52€*	-27.36 (24.23)
*Pharmacy limit Up to 61.75€*	193.29 (140.03)
*40% co-payment*	-75.15 (10.41)
*50% co-payment*	-103.86 (10.96)
*60% co-payment*	-157.43 (19.27)
*Excluded from co-payment*	-309.61 (15.42)

^a^, ^b^ and ^c^ denote statistical significance levels 10%, 5% and 1%, respectively. Standard errors are in parenthesis. The sample size is 91,286, and basic health areas' fixed effects were included as additional covariates

## Abbreviations

ADHD: Attention-Deficit/Hyperactivity Disorder
DRG: Diagnostic Related Group
FMM: Finite Mixture Models
GLM: Generalised Linear Models
GPs: General Practitioners
HCP: Health Care Procedures
ICD-9-CM: The International Classification of Diseases, 9^th^ revision, Clinical Modification
IV: Instrumental Variables
MPR: Medication Possession Ratio
PDC: Proportion of Days Covered
DSM: Diagnostic and Statistical Manual of Mental Disorders
